# Impaired cardiac and skeletal muscle bioenergetics in children, adolescents, and young adults with Barth syndrome

**DOI:** 10.14814/phy2.13130

**Published:** 2017-02-14

**Authors:** Adil Bashir, Kathryn L. Bohnert, Dominic N. Reeds, Linda R. Peterson, Adam J. Bittel, Lisa de las Fuentes, Christina A. Pacak, Barry J. Byrne, W. Todd Cade

**Affiliations:** ^1^ Department of RadiologyWashington University School of MedicineSt. LouisMissouri; ^2^ Department of Electrical and Computer EngineeringAuburn UniversityAuburnAlabama; ^3^ Program in Physical TherapyWashington University School of MedicineSt. LouisMissouri; ^4^ Department of MedicineWashington University School of MedicineSt. LouisMissouri; ^5^ Department of PediatricsUniversity of FloridaGainesvilleFlorida

**Keywords:** Barth syndrome, energetics, exercise, mitochondria, muscle

## Abstract

Barth syndrome (BTHS) is an X‐linked condition characterized by altered cardiolipin metabolism and cardioskeletal myopathy. We sought to compare cardiac and skeletal muscle bioenergetics in children, adolescents, and young adults with BTHS and unaffected controls and examine their relationships with cardiac function and exercise capacity. Children/adolescents and young adults with BTHS (*n* = 20) and children/adolescent and young adult control participants (*n* = 23, total *n* = 43) underwent ^31^P magnetic resonance spectroscopy (^31^P‐MRS) of the lower extremity (calf) and heart for estimation of skeletal muscle and cardiac bioenergetics. Peak exercise testing (VO_2peak_) and resting echocardiography were also performed on all participants. Cardiac PCr/ATP ratio was significantly lower in children/adolescents (BTHS: 1.5 ± 0.2 vs. Control: 2.0 ± 0.3, *P* < 0.01) and adults (BTHS: 1.9 ± 0.2 vs. Control: 2.3 ± 0.2, *P* < 0.01) with BTHS compared to Control groups. Adults (BTHS: 76.4 ± 31.6 vs. Control: 35.0 ± 7.4 sec, *P* < 0.01) and children/adolescents (BTHS: 71.5 ± 21.3 vs. Control: 31.4 ± 7.4 sec, *P* < 0.01) with BTHS had significantly longer calf PCr recovery (*τ* PCr) postexercise compared to controls. Maximal calf ATP production through oxidative phosphorylation (Qmax‐lin) was significantly lower in children/adolescents (BTHS: 0.5 ± 0.1 vs. Control: 1.1 ± 0.3 mmol/L per sec, *P* < 0.01) and adults (BTHS: 0.5 ± 0.2 vs. Control: 1.0 ± 0.2 mmol/L sec, *P* < 0.01) with BTHS compared to controls. Blunted cardiac and skeletal muscle bioenergetics were associated with lower *V*O_2peak_ but not resting cardiac function. Cardiac and skeletal muscle bioenergetics are impaired and appear to contribute to exercise intolerance in BTHS.

## Introduction

Barth syndrome (BTHS) is a X‐linked disorder resulting from mutations in the gene encoding for tafazzin (TAZ) (Bione et al. 1996) and is characterized by heart failure, exercise intolerance, fatigue, and premature mortality (Barth et al. 1983). TAZ, a phospholipid‐lysophospholipid transacylase, remodels monolysocardiolipin to tetralinoleoyl‐cardiolipin which provides inner membrane stability necessary for ATP production by the electron transport chain (Zhang et al. 2002). Mutations in TAZ result in an increase in monolysocardiolipin and reduction in tetralinoleoyl‐cardiolipin (Schlame et al. 2003), smaller and fragmented mitochondria (Wang et al. 2014), disruptions in mitochondrial supercomplexes (Xu et al. 2016), instability in the mitochondrial inner membrane (Zhang et al. 2002), and markedly reduced respiratory capacity (Wang et al. 2014).

Our group previously demonstrated severe impairments in whole‐body oxygen consumption during peak exercise that was due to a combination of cardiac and skeletal muscle impairments (Spencer et al. 2011). Furthermore, using near‐infrared spectroscopy we found that skeletal muscle (lateral quadriceps) oxygen extraction was significantly blunted during exercise in children, adolescents, and young adults with BTHS suggesting impaired mitochondrial energetics in these individuals (Spencer et al. 2011). Wang et al. (2014). recently found blunted mitochondrial energetics and reduced contractile function in cardiomyocytes derived from inducible pluripotent stem cells made from human BTHS fibroblasts; however, the effects of BTHS on whole tissue cardiac and skeletal muscle bioenergetics and their relationship with cardiac and skeletal muscle function in humans, in vivo, remain unknown.

The use of phosphorus magnetic resonance spectroscopy (^31^P‐MRS) to noninvasively measure skeletal muscle (Kemp et al. 2015; Sapega et al. 1993) and cardiac bioenergetics (Hudsmith and Neubauer 2009; Ingwall et al. 1985) is well‐established (Chance et al. 1981). Measurement of phosphocreatine (PCr) recovery following muscular exercise provides an accurate estimate of oxidative metabolism (Meyer 1988) as PCr intricately links mitochondrial and myofibrillar ATP production from oxidative phosphorylation (Altschuld and Brierley 1977; Meyer et al. 1984). Reduced PCr/ATP ratio is characteristic of failing myocardium (Conway et al. 1991) and represents a reduced capacity or reserve to provide ATP to the working heart, especially during stressed conditions (Nascimben et al. 1996; Smith et al. 2006). Skeletal muscle and cardiac bioenergetics might be useful biomarkers or outcome measures for prospective studies and clinical trials in BTHS as individuals with BTHS frequently have normal resting cardiac function without a predictable natural history (Spencer et al. 2006; Thompson et al. 2016). In addition, cardiac bioenergetics have been shown to respond favorably to interventions (Spoladore et al. 2013) and to be useful predictors of cardiovascular and all‐cause mortality in patients with non‐BTHS heart failure (Neubauer et al. 1997).

Therefore, the primary objective of this study was to compare cardiac and skeletal muscle bioenergetics in children, adolescents, and young adults with BTHS to unaffected age and activity level matched controls. The secondary objective was to examine the relationships between cardiac and skeletal muscle bioenergetics, cardiac function, and exercise capacity. We hypothesized that individuals with BTHS would have marked reductions in cardiac and skeletal muscle bioenergetics and that these would be associated with lower cardiac function and exercise capacity.

## Materials and Methods

Forty‐three (total *n* = 43) participants: 30 children/adolescents (BTHS *n* = 14, Control *n* = 16) and 13 adults (BTHS *n* = 6, Control *n* = 7) between 18 and 36 years of age were included (Table 1). Participants with BTHS were recruited through the Barth Syndrome Registry located at the University of Florida and controls were recruited through nonaffected siblings and local recruitment from the Greater St. Louis community. Control participants were considered sedentary, defined as currently participating in structured exercise ≤2× per week. All participants completed skeletal muscle ^31^P‐MRS, however, due to technical difficulties two children and one adult were not able to complete the cardiac ^31^P‐MRS. Studies were approved by the Human Studies Committee at Washington University in St. Louis and all participants provided assent and all participants and/or parents provided written informed consent.

**Table 1:** Participant demographics, peak exercise testing, and echocardiography

	Control (*n* = 16)	BTHS (*n* = 14)	*P* ‐value	Control (*n* = 7)	BTHS (*n* = 6)	*P* ‐value
Age (years)	12 ± 3	13 ± 3	0.36	23 ± 5	27 ± 4	0.10
Height (cm)	154.5 ± 17.7	143.8 ± 14.2	0.08	175.7 ± 4.7	178.4 ± 6.7	0.39
Weight (kg)	47.3 ± 18.6	34.9 ± 12.4	0.04	84.5 ± 14.4	68.0 ± 12.6	0.02
FFM (kg)	38.9 ± 15.3	24.3 ± 6.7	0.003	65.7 ± 8.4	42.2 ± 7.2	<0.001
FFM (%)	83 ± 7	68 ± 13	<0.001	79 ± 8	64 ± 11	0.01
FM (kg)	8.0 ± 5.7	12.8 ± 10.2	0.12	18.5 ± 9.3	25.3 ± 9.8	0.17
FM (%)	17 ± 7	32 ± 13	<0.001	21 ± 8	36 ± 11	0.01
Resting HR (bpm)	83 ± 7	85 ± 11	0.58	75 ± 19	74 ± 16	0.43
Resting SBP (mmHg)	106 ± 12	101 ± 12	0.28	137 ± 6	104 ± 9	<0.001
Resting DBP (mmHg)	65 ± 10	67 ± 9	0.58	84 ± 9	65 ± 10	0.17
Exercise variables						
*V*O_2peak_ (mL/kg/min)	36.1 ± 7.7	13.7 ± 3.2	<0.001	35.7 ± 5.9	12.3 ± 3.5	<0.001
Peak watts	143 ± 70	39 ± 10	<0.001	239 ± 51	61 ± 11	<0.001
Peak RER	1.1 ± 0.1	1.3 ± 0.2	0.002	1.2 ± 0.1	1.5 ± 0.2	<0.001
Peak HR	184 ± 11	158 ± 22	<0.001	186 ± 15	159 ± 11	0.001
Peak SBP (mmHg)	144 ± 19	116 ± 16	<0.001	202 ± 24	137 ± 16	<0.001
Peak DBP (mmHg)	70 ± 11	74 ± 16	0.47	88 ± 11	84 ± 11	0.70
Echocardiography						
LVM (g)	94.6 ± 30.1	74.6 ± 28.6	0.13	149.7 ± 37.8	127.2 ± 25.6	0.29
Ejection fraction (%)	67 ± 4	63 ± 5	0.02	64 ± 5	57 ± 15	0.18
Fractional shortening (%)	40 ± 6	34 ± 11	0.08	36 ± 6	30 ± 8	0.05
Global strain (%)	−21 ± 3	−20 ± 2	0.25	−18 ± 2	−15 ± 3	0.05

Values are means ± SD.

FFM, fat‐free mass; FM, fat mass; *V*O_2peak_, volume of oxygen consumption during peak; LVM, left ventricular mass.

### Cardiac bioenergetics

Cardiac and skeletal muscle experiments were performed by using a Siemens 3T magnet equipped with broadband capabilities (Siemens Medical Systems, Erlangen, Germany) using a home built ^31^P transmit‐receive RF coil. Cardiac ^31^P‐MRS was performed with the participant in the supine position using 1D‐ISIS localization, as previously described (Bashir and Gropler 2014). Briefly, participants were positioned with their heart at the center of the magnet, and the ^31^P surface coil was positioned on the chest with the center of the coil just below the mitral valve of the heart using proton scout images. A small fiducial marker placed at the center of the RF coil to adjust the positioning of the coil relative to the heart. A non‐localized ^31^P spectrum was then acquired and RF transmit frequency was centered on the PCr resonance. Data were acquired following 2.56 msec adiabatic half passage pulse radiofrequency pulses applied at 6 s intervals and with a spectral width of 2 kHz and 64 averages per spectrum.

Spectra were processed offline using the jMRUI (Java‐Based Magnetic Resonance User Interface) software (Naressi et al. 2001). Spectra were fitted in time domain by using a nonlinear least‐squares algorithm (AMARES) (Vanhamme et al. 1997). ATP, PCr, 2,3‐diphosphoglycerate (DPG), and phosphodiester (PDE) signals were fitted to Lorentzian line shapes. The three ATP peaks were fitted as two doublets and one triplet, with equal amplitudes and line widths and prior knowledge for the J‐coupling constant. After fitting, the *γ*‐ATP peak area was corrected for blood contamination according to the amplitude of the 2,3‐diphosphoglycerate (2,3‐DPG) peak as described previously. PCr/ATP ratio was calculated and corrected for partial saturation (El‐Sharkawy et al. 2009).

### Skeletal muscle bioenergetics

Calf muscle bioenergetic function was determined from the recovery of PCr after plantar flexion exercise. Single‐leg plantar flexion exercise was performed using a custom‐built ergometer designed to fit in the bore of the MRI scanner. During the exercise protocols, participants laid supine on the ergometer with the legs extended and strapped to the ergometer base in order to reduce bulk motion. A 9 cm ^31^P RF coil was placed under the calf and held in place with straps. Exercise consisted of 0–22° plantarflexion exercise against a force adjusted to 40% of maximal voluntary contraction (MVC) using a pressurized pneumatic cylinder. To ensure that all participants were capable of performing plantar flexions inside the scanner they were asked to perform (10 or less) plantar flexion extensions while in the scanner. Contraction‐relaxation cycles were gated to the magnetic resonance acquisition on the basis of an audible signal with one contraction every 2 sec. After the initial familiarization period the participants were given a 10 min rest before the start of the exercise‐recovery ^31^P‐MRS experiment. During the exercise, each participant performed 30 plantar flexions at a cadence of 1 plantar flexion exercise every 2 sec acoustically guided by gradient noise from the scanner. Exercise performance was monitored and recorded using Digital Gauge and Transmitter (Ashcroft Industries, Stratford, CT) and USB‐6009 Data Acquisition unit (National Instruments, Austin, TX).

^31^P‐MRS data were also acquired at rest with repetition time of 20 sec and 2 sec to correct for partial saturation. The exercise protocol was then commenced and data were acquired during and for 8 min after the end of the exercise protocol. Pulse parameters were as follows: 2.56 msec adiabatic half passage pulse, 2 sec repetition time 1024 data points, 2.5 kHz spectral width.

Relative ratio of the resonances area was determined using jMRUI software as described above. Absolute concentrations of ^31^P metabolites were calculated assuming that resting [ATP] is 8.2 mmol/L (Kemp et al. 2007). Intramuscular pH was calculated from the chemical shift difference between PCr and Pi resonances in parts per million using the following relationship (Taylor et al. 1983)

$$\text{pH}=6.75+\log\frac{\sigma -3.27}{5.96-\sigma }$$

ADP concentration was calculated from [PCr] and pH using the creatine kinase equilibrium constant (*K*_*eq*_ = 1.66 × 10^9^ M^−1^) and assuming that total creatine [PCr+Cr] is 42.5 mmol/L (Kemp et al. 2001,2007).

$$\left[\text{ADP}\right]=\frac{\left[\text{ATP}][\text{Cr}\right]}{\left[\text{PCr}\right]\left[H^{+}\right]K_{\mathrm{eq}}}$$

Oxidative capacity was determined from recovery of PCr after exercise. Postexercise recovery of PCr was fitted to a single exponential curve $\text{PCr}\left(t\right)=\Delta \text{PCr}\left(1-e^{-t/\tau \mathrm{PCr}}\right)+{\text{PCr}}_{\mathrm{end}}$ where PCr_end_ is the concentration of PCr at the end of exercise and ΔPCr is the PCr consumed during exercise (resting [PCr] – PCr_end_). The initial rate of PCr recovery (V_iPCr_) was calculated by

$$V_{i\mathrm{PCr}}=\frac{\Delta \text{PCr}}{\tau_{\mathrm{PCr}}}$$

The maximum oxidative capacity was calculated according Michaelis–Menten kinetics with *K*_*m*_ = 30 *μ*mol/L and taking into account the ADP concentration at the end of exercise [ADP]_end_ and the initial rate of PCr recovery (V_iPCr_) (Chance et al. 1985; Kemp and Radda 1994; Layec et al. 2011)

$$Q_{\max-\mathrm{ADP}}=V_{i\text{PCr}}\left(1+\frac{K_{m}}{{\left[\text{ADP}\right]}_{\mathrm{end}}}\right)$$

Maximal oxidative flux was also calculated according to the linear model (Meyer 1988)

$$Q_{\max-\mathrm{lin}}=\frac{\left[\text{PCr}\right]}{\tau_{\mathrm{PCr}}}$$

Rate of oxidative mitochondrial ATP synthesis was based on the hyperbolic relationship between the oxidative ATP synthesis rate and the free cytosolic ADP concentration

$${\text{ATP}}_{ox}=\frac{Q_{\max-\mathrm{ADP}}}{1+K_{m}/\left[\text{ADP}\right]}$$

This model assumes that the oxygen delivery to mitochondria is not limited which is expected due to the nature of the exercise protocol (Lanza et al. 2005; Layec et al. 2011).

### Body composition

Fat mass (kg and %) and fat‐free mass (kg and %) in all participants were determined through air‐displacement plethymosgraphy (Bod Pod, Life Measurements Inc., Concord, CA).

### Echocardiography

All participants underwent conventional two‐dimensional (2‐D) and pulsed‐wave Doppler, (General Electric Vivid E9; Waukesha, WI). Left ventricular (LV) mass was determined by 2‐D echocardiography according to recommendations of the American Society of Echocardiography (Lang et al. 2005). LV end‐diastolic and end‐systolic volumes were determined using the method of disks and LV ejection fraction was calculated. LV end‐diastolic and end‐systolic dimensions (LVED and LVES) were measured in the parasternal long‐axis view. Fractional shortening was calculated as (LVED‐LVES)/LVED. 2D speckle tracking echocardiographic‐derived peak systolic strain was determined from the apical four‐chamber, two‐ chamber, and apical long‐axis views and calculated as the average of the basal, mid‐ and apical segments of all walls.

### Graded exercise testing

All participants performed a graded exercise test on an electronically braked recumbent cycle ergometer (Lode Corvial, The Netherlands). Work rate on the cycle ergometer started at 10 watts (W) and increased 10 W/min for all BTHS and for unaffected child controls and started at 20 W and increased 20 W/min for adolescent and young adult controls until volitional exhaustion. 12‐lead ECG, blood pressure, and volume of oxygen (*V*O_2_) consumption, carbon dioxide (*V*CO_2_) production, and ventilation (ParvoMedics TrueOne, Sandy, UT) were continuously measured. Exercise testing was considered peak with attainment of ≥85% predicted peak heart rate (220‐age) and/or RER ≥ 1.10 according to the (American College of Sports Medicine (2000)).

### Statistical analysis

Results are presented as mean ± standard deviation. Student's unpaired *t*‐tests were used to compare the variables between BTHS and controls. The relationships between variables were analyzed by the Pearson correlation coefficient as data were normally distributed. Statistical significance was accepted at *P* < 0.05

## Results

### Demographics

Participant demographics are provided in Table 1. There was one African American adult participant with BTHS, one African American adult control, two Asian adult controls, and the remaining participants were Caucasian. Participants with BTHS were currently taking: beta‐blockers (52%), ACE inhibitors (39%), cardiac glycosides (35%), gram colony stimulating factor (30%), antidepressants (17%), and supplemental amino acids (39%). Age and height were not different between groups. Weights in children/adolescents and adults with BTHS were significantly lower than controls. Absolute fat‐free mass (kg) and fat‐free mass expressed as % of total mass in children/adolescents and adults with BTHS were significantly lower than controls. Absolute fat mass was not different between groups, however, % fat mass, was higher in both children/adolescents and adults with BTHS compared to control groups.

### Peak exercise testing

*V*O_2peak_ and peak cycle ergometer resistance (watts) were significantly lower in children/adolescents and adults with BTHS compared to control groups. Peak respiratory exchange ratio was higher in BTHS versus controls and peak heart rate was lower in both children/adolescents and adults with BTHS compared to control groups (all Table 1).

### Echocardiography

Ejection fraction and fractional shortening were lower in children/adolescents with BTHS versus controls. Fractional shortening was lower in adults with BTHS versus controls but ejection fraction was not different between groups. Global strain was lower in adults with BTHS versus controls, however, strain was not different between the two groups of children/adolescents (all Table 1).

### Cardiac bioenergetics

**Figure 1:**
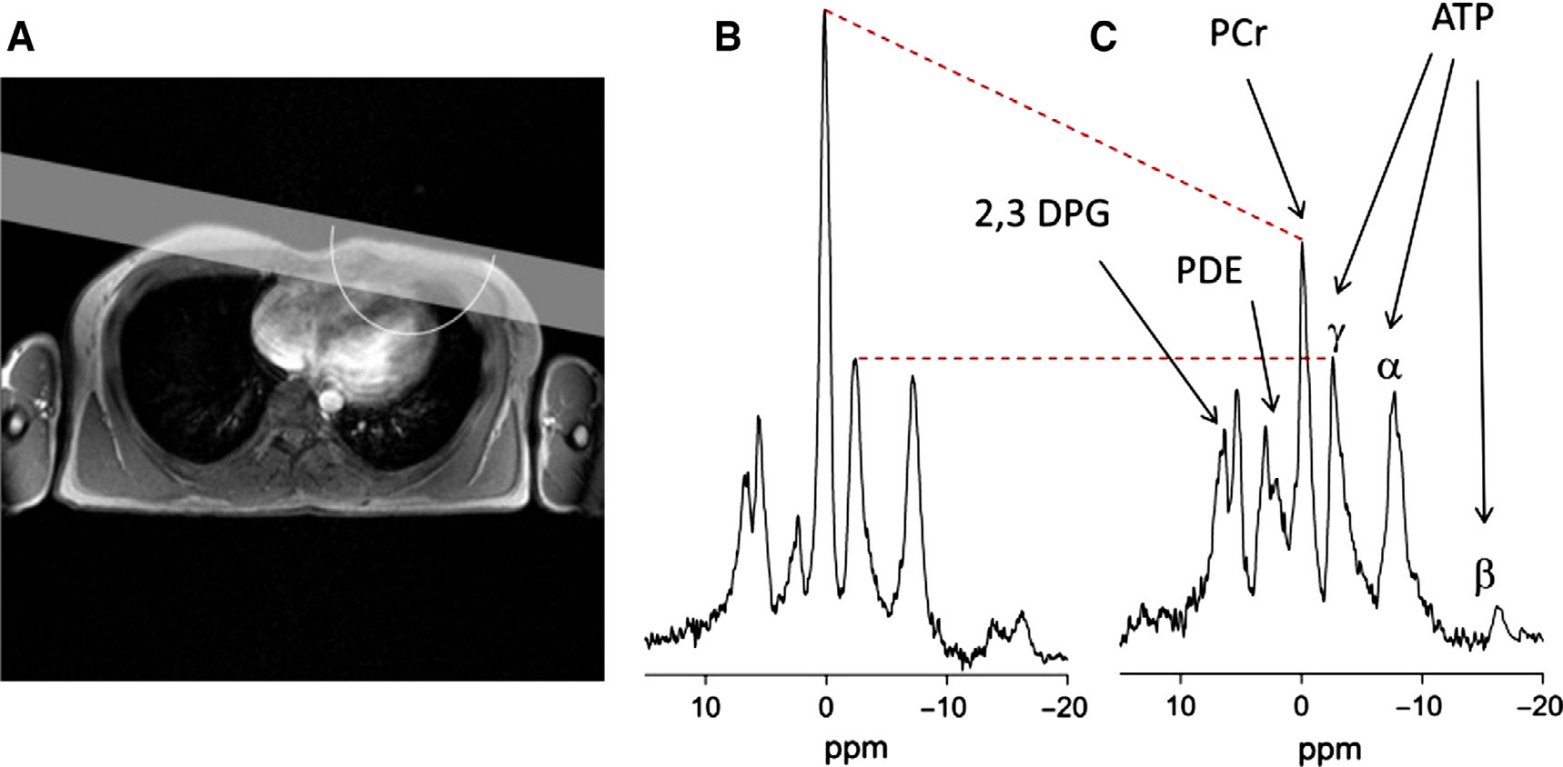
Reference images of the heart. Location of saturation band and approximate sensitive region of the RF coil is also shown (A) Spectrum from a healthy control (B) and a participant with Barth syndrome (C) detailing the peaks of PCr, ATP, PDE, and 2,3 DPG. PCr resonance amplitude is reduced in spectrum from Barth participant with similar ATP amplitudes. The red dashed lines provide a visual cue of changes in PCr and ATP peaks. *β*‐ATP resonance in the spectra is diminished due to the bandwidth limitations of the adiabatic RF pulse. DPG, 2,3‐diphosphoglycerate; PDE, phosphodiester.

A sample reference image of the heart and location of saturation band to eliminate the signal from the chest muscles is shown in Figure 1A. A typical example of cardiac ^31^P‐MR spectra from a unaffected control participant (Fig. 1B) and a participant with BTHS (Fig. 1C) with reduced PCr amplitude is provided in Figure 1. Cardiac PCr/ATP ratio was significantly lower in children/adolescents and adults with BTHS compared to control groups (Table 2).

**Table 2:** Resting cardiac and skeletal muscle bioenergetics

	Children			Adults		
Skeletal Muscle	Control (*n* = 16)	BTHS (*n* = 14)	*P* ‐value	Control (*n* = 7)	BTHS (*n* = 6)	*P* ‐value
pH	7.0 ± 0.0	7.1 ± 0.1	0.45	7.0 ± 0.0	7.0 ± 0.0	0.48
[PCr] (mmol/L)	31.3 ± 1.9	35.8 ± 1.8	<0.01	33.3 ± 2.5	35.8 ± 2.5	0.11
[Pi] (mmol/L)	3.0 ± 0.7	2.3 ± 1.2	0.06	2.8 ± 0.7	2.0 ± 0.8	0.08
[PCr]/[Pi]	11.0 ± 3.5	18.7 ± 7.5	<0.01	13.2 ± 5.2	22.1 ± 11.9	0.10
[PDE] (mmol/L)	1.7 ± 0.7	2.6 ± 0.9	0.01	2.4 ± 0.8	2.9 ± 0.7	0.35
[ADP]*μ*mol/L	19.2 ± 4.3	10.6 ± 3.4	<0.01	14.8 ± 5.6	9.7 ± 4.1	0.09
Cardiac						
[PCr]/[ATP]	2.0 ± 0.2	1.5 ± 0.2	<0.01	2.3 ± 0.2	1.9 ± 0.2	<0.01

Values are means ± SD.

PCr, phosphocreatine concentration; Pi, inorganic phosphate concentration; PDE, phosphodiesterase concentration; ADP, adenosine diphosphate concentration; ATP, adenosine triphosphate concentration.

### Skeletal muscle bioenergetics

#### Resting

At rest, pH was not different between the two groups in both children/adolescents and adults (Table 2). [PCr], [PCr]/[Pi] ratio, and [PDE] were significantly higher in children/adolescents with BTHS versus controls and [Pi] and [ADP] tended to be lower in both children/adolescents and adults with BTHS as compared to controls. [PCr] tended to be lower and [PCr]/[Pi] ratio tended to be higher in adults with BTHS versus controls.

### Exercise recovery

**Figure 2:**
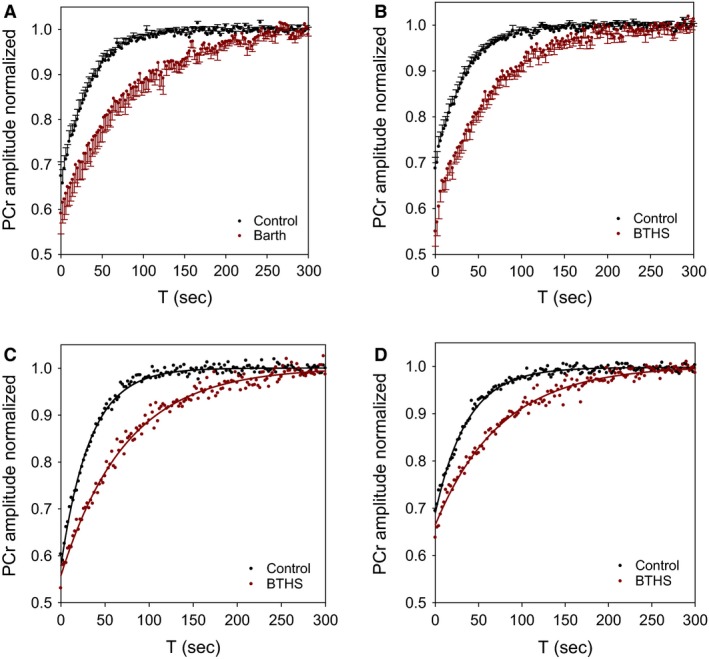
Typical postexercise PCr recovery data. PCr amplitude (normalized to resting condition) for all participants (A) Adults, (B) Children/Adolescents). Error bars represent standard error. Representative PCr recovery data from adults (C) and children/adolescents (D) showing example of signal exponential fit.

A typical spectrum recorded at rest from calf muscle is displayed in Figure 2A and MR spectra that was continuously recorded during the standardized rest‐exercise‐recovery protocol (Fig. 2B). As expected, intramuscular pH became briefly alkaline during exercise, however, the end‐exercise pH was similar to baseline. PCr decreased during exercise and increased rapidly after cessation of exercise and followed a mono‐exponential time course. ATP concentration, determined from the *γ*‐peak, did not differ across rest, exercise, or recovery for both protocols. The calculated ADP concentration increased above baseline during exercise in both groups and remained significantly higher at end‐exercise in children with BTHS (data not shown). Adults and children/adolescents with BTHS had significantly longer *τ*_PCr_ compared to controls. Initial rate of PCr recovery (Vi) was significantly lower in children/adolescents and adults with BTHS versus controls. Maximal ATP production rates through oxidative phosphorylation estimated by Q_max‐lin_ and Q_max‐ADP_ and ATP_ox_ was significantly lower in children/adolescents and adults with BTHS versus control groups (Table 3). There were no differences in skeletal muscle MRS exercise variables between adults versus children/adolescents in with BTHS or in Controls.

**Figure 3:**
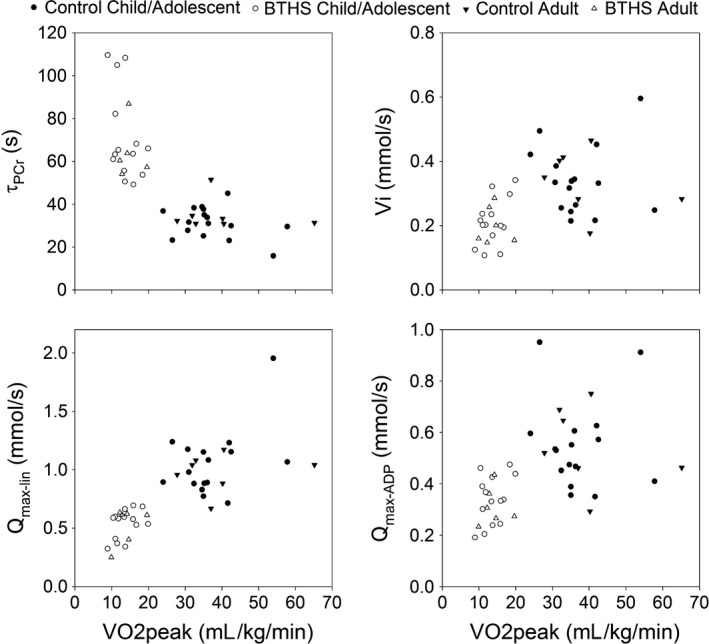
Scatterplots of *V*O_2peak_ and (A) *τ* PCR, (B) Vi, C) Qmax‐lin, and (D) Qmax‐ADP during 60‐sec exercise.

**Table 3:** Skeletal muscle bioenergetics during/postexercise

	Children			Adults		
	Control (*n* = 16)	BTHS (*n* = 14)	*P* ‐value	Control (*n* = 7)	BTHS (*n* = 6)	*P* ‐value
pH end	7.0 ± 0.1	7.0 ± 0.1	0.60	7.0 ± 0.1	7.0 ± 0.2	0.73
[ADP] end (*μ*mol/L)	51.7 ± 14.3	54.9 ± 24.3	0.70	49.5 ± 6.4	59.6 ± 23.8	0.30
T_PCr_ (sec)	31.4 ± 7.3	71.5 ± 21.3	<0.01	35.0 ± 7.4	76.4 ± 31.6	<0.01
Vi (mmol/L per sec)	0.3 ± 0.1	0.2 ± 0.1	<0.01	0.3 ± 0.1	0.2 ± 0.1	<0.01
Q_max‐lin_ (mmol/L per sec)	1.1 ± 0.3	0.5 ± 0.1	<0.01	1.0 ± 0.2	0.5 ± 0.2	<0.01
Q_max‐ADP_ (mmol/L per sec)	0.5 ± 0.2	0.3 ± 0.1	<0.01	0.5 ± 0.2	0.3 ± 0.1	<0.01
ATPox (mM/L per min)	20.6 ± 5.7	12.7 ± 4.5	<0.01	20.0 ± 6.0	12.0 ± 3.3	0.02

Values are means ± SD.

ADP, adenosine diphosphate concentration; T_PCr_, Tau phosphocreatine; *V*i, initial rate of PCr recovery; Q_max‐lin_, Q maximum linear equation; Q_max‐ADP_, Q maximum ADP equation, ADP, adenosine diphosphate concentration, BTHS, Barth syndrome.

### Relationships between cardiac and muscle bioenergetics, cardiac function, and exercise capacity

Examining both sets of participants with BTHS and controls (*n* = 43), ^31^P‐MRS markers of mitochondrial function (*τ*_PCr_ (*r* = −0.72)_,_ Vi (*r* = 0.46), Q_max‐lin_ (*r* = 0.69), Q_max‐ADP_ (*r* = 0.46), all *P* < 0.01) revealed significant correlations with *V*O_2peak_. Cardiac PCr/ATP was also associated with *V*O_2peak_ (*r* = 0.60, *P* < 0.001) but was not associated with resting cardiac function (ejection fraction, fractional shortening, global strain). When examining participants with BTHS only (*n* = 20), *τ*_PCr_ (*r* = −0.51, *P* = 0.02) was related to *V*O_2peak_ and Q_max‐lin_ (*r* = 0.41, *P* = 0.07) tended toward being related to *V*O_2peak_. Cardiac PCr/ATP was not associated with *V*O_2peak_ or any measurement of cardiac function at rest.

## Discussion

The principal and novel findings of this study were that skeletal muscle and cardiac bioenergetics, as determined from postexercise PCr recovery kinetics using ^31^P‐MRS, were impaired in both children/adolescents and young adults with BTHS when compared to unaffected, age‐matched, sedentary controls. A secondary novel finding was that cardiac and skeletal muscle bioenergetic impairments were associated with exercise intolerance in this cohort. Mitochondrial function in tissues such as myocardium and skeletal muscle is closely integrated with physiological demands below the anaerobic threshold (Kemp et al. 2015; Wasserman 1987). In BTHS, in vitro mitochondrial respiration has been shown to be impaired in inducible pluripotent stem cell derived human cardiomyocytes (Wang et al. 2014); our data extend this finding by demonstrating altered in vivo mitochondrial function in humans with BTHS. Possible mechanisms for BTHS‐associated mitochondrial dysfunction include mitochondrial supercomplex destabilization (Gonzalvez et al. 2013), higher degradation and reduced levels of mitochondrial cardiolipin (Xu et al. 2016), abnormal mitochondrial morphology (Barth et al. 1983; Bissler et al. 2002), excessive production of reactive oxygen species (Wang et al. 2014), and/or defects in ATP synthase activity (Wang et al. 2014). In this study, skeletal muscle bioenergetics were not different between adults and children/adolescents in either BTHS or unaffected controls. This appears to be in contrast to studies in healthy children and adults as children were found to rely more on oxidative metabolism during exercise than adults (Ratel et al. 2008; Tonson et al. 2010).

Cardiomyopathy with left ventricular noncompaction is a hallmark feature of BTHS (Barth et al. 1983; Spencer et al. 2006). However, the majority of individuals with BTHS have normal to low‐normal systolic function at rest (Spencer et al. 2006) that can unpredictably decline and improve throughout childhood, adolescence and young adulthood (Thompson et al. 2016). In this study, we found impaired cardiac bioenergetic reserve (i.e., PCr/ATP) in both children/adolescents and adults with BTHS. This is in contrast to PCr/ATP data in one infant with BTHS presented in the supplementary material of a recent study (Wang et al. 2014). Also, PCr/ATP was greater in adults versus children/adolescents in both BTH and unaffected controls. The reason for these differences remain unclear; one previous study found that children rely more on oxidative metabolism versus creatine kinase reaction than adults (Tonson et al. 2010), however, these data were obtained from skeletal muscle. The PCr energy system provides a reserve capacity for cardiac and skeletal muscle to provide inorganic phosphate to adenosine diphosphate (ADP) in suprabasal energy requiring states (Kemp et al. 2015; Neubauer et al. 1997). Although many participants with BTHS have normal or low normal systolic function, the ability to increase myocardial contractility during high energy requiring conditions (i.e., exercise) is impaired (Spencer et al. 2011). Thus the reduced cardiac energetic reserve in children/adolescents and young adults seen in this study might explain the inability to increase myocardial contractility during exercise and partially explain the resultant exercise intolerance that is characteristic of BTHS. PCr/ATP might be a useful prognostic factor in BTHS, even in the absence of resting left ventricular dysfunction, as PCr/ATP has been shown to be a better predictor of cardiovascular mortality than ejection fraction or heart failure classification in patients with non‐BTHS heart failure (Neubauer et al. 1997).

Exercise intolerance has been previously demonstrated in both the BTHS‐mouse model (i.e., TAZ knockdown) (Powers et al. 2013) and in humans with BTHS (Cade et al. 2016; Spencer et al. 2011); our current data confirm these findings. Our earlier study found that exercise intolerance (i.e., diminished *V*O_2peak_) was mediated by both cardiac and skeletal muscle impairments (Spencer et al. 2011). Specifically, we found that skeletal muscle oxygen extraction measured by near‐infrared spectroscopy (NIRS) and systolic function was markedly blunted during exercise in adolescents and young adults with BTHS compared to unaffected, age‐matched controls. Our data from this study expand these findings and demonstrates that impaired cardiac and skeletal muscle tissue bioenergetics are associated with blunted whole‐body oxygen consumption during exercise. Although skeletal muscle mitochondrial function and whole‐body oxygen consumption are tightly associated and this relationship should be expected (Larson‐Meyer et al. 2000), this finding helps correlate tissue‐level bioenergetic impairments with functional outcomes; a link not previously demonstrated in humans with BTHS.

Our study found higher resting skeletal muscle [PCr]/[Pi] in children/adolescents with BTHS and a tendency for higher [PCr]/[Pi] in adults with BTHS when compared with unaffected controls. Higher [PCr]/[Pi] is frequently observed in muscles with large fraction of Type 2 (fast twitch) glycolytic fibers and the opposite is true in muscles with a large volume fraction of Type 1 (oxidative) fibers (Kushmerick et al. 1992; Takahashi et al. 1996). Although this study did not measure the fraction of Type 1 versus Type 2 muscle fibers in participants, previous data suggest that individuals with BTHS rely on glycolytic metabolism to a greater extent than those without BTHS, likely due to an impairment in fat oxidation. Specifically, adolescents/young adults with BTHS had a higher respiratory exchange ratio during exercise (Spencer et al. 2011) and a greater glucose rate of disposal during a hyperinsulinemic‐euglycemic clamp procedure (Cade et al. 2013); both suggestive of higher glycolytic dependence. If true, this higher glycolytic capacity might be compensatory for an impaired capacity to generate ATP via oxidative phosphorylation as seen in this study.

We also found significantly higher resting skeletal muscle phosphodiester (PDE) levels in children with BTHS when compared to unaffected controls. PDE is an intermediate of membrane phospholipid degradation, may be related to membrane proliferation and remodeling (Waters et al. 2003), and is elevated in other mitochondrial myopathies (Edwards et al. 1982; Matthews et al. 1991). Elevated PDE has been associated with sarcolemmal damage, muscle atrophy, and fiber loss (Taylor et al. 1997), potentially resulting from excessive reactive oxygen species production (Lanza and Nair 2010). Although not measured in this study, sarcolemmal disorganization has been previously shown in inducible pluripotent stem cell derived BTHS‐human cardiomyocytes that was improved with linoleic acid supplementation that resulted in a reduction in reactive oxygen species production (Wang et al. 2014). Since mitochondria contribute to the regulation of sarcomere formation during cardiomyocyte maturation (Hom et al. 2011), PDE concentration via ^31^P‐MRS might be a useful biomarker for skeletal muscle sarcomere and mitochondrial membrane integrity assessed through potential interventions.

There are some limitations of our study. Participants with BTHS had lower fat‐free mass (i.e., skeletal muscle) which might contribute to worse PCr kinetics, however; postexercise PCr recovery kinetics has been shown to be independent of muscle mass (Minotti et al. 1990). Participants with BTHS had higher [PCr]/[Pi] which could potentially bias the calculation of Q_max‐lin_ on the basis of the resting [PCr] (Meyer 1988; Tonson et al. 2010), but we found that *Q*_max‐ADP_ (which is independent of such bias (Layec et al. 2011)) was also significantly reduced in participants with BTHS. PCr initial rate of recovery (Vi) depends on ATP turnover during exercise and can be affected by exercise intensity (i.e., work rate) (Roussel et al. 2000). Although in this study work rate was normalized via 1‐repetition maximum testing, there might have been interparticipant differences in exercise work rate therefore affecting Vi. Although unaffected controls reported a sedentary lifestyle, it is possible that participants with BTHS were more inactive than their peers and group differences in physical activity could have affected the results. However, given the very large group differences in mitochondrial energetics this was not likely to be solely due to differences in physical activity level. Also, several participants with BTHS were taking cardiac supportive care therapy including beta‐blockers, ACE inhibitors and cardiac glycosides. Beta‐blocker therapy appears to improve mitochondrial function through increase mitochondrial biogenesis and protection from oxidative stress (Gomez et al. 2014; Sgobbo et al. 2007; Yao et al. 2016). The effects of ACE inhibitor therapy on mitochondrial function are not clear: some studies demonstrating a protective effect from physiologic insult (e.g., myocardial infarction) (Hiona et al. 2011; Zoll et al. 2006) where other report no significant effect on mitochondrial function, exercise capacity, or protection from insult (Bahi et al. 2004; Thaveau et al. 2010). Cardiac glycosides appear to have a minimal effect on mitochondrial function in the absence of toxicity (Liu et al. 2010). Taking these data in sum, the effect, if any, of cardiac supportive therapy on mitochondrial function would have likely been beneficial and would have reduced the differences between groups; making the comparison more conservative. Lack of biological quantification of [PCr] and [ATP] concentrations is a limitation of this study. Muscle biopsy could have provided these data (and other mitochondrial morphologic information), however, biopsy is an invasive procedure which makes its use difficult in pediatric populations and especially in those prone to bacterial infections such as BTHS. Thus ^31^PMRS provides a viable approach for this population and longitudinal assessments.

## Conclusion

In summary, we found altered cardiac and skeletal muscle (calf) bioenergetics in children/adolescents and young adults with BTHS that were associated with exercise intolerance. ^31^P‐MRS is a useful technique that can quantify mitochondrial impairments in BTHS and might be an important biomarker or outcome measure in future intervention studies.

## Conflict of Interests

None of the authors in the manuscript have any financial conflicts of interest to report.
